# Two-stage family-based designs for sequencing studies

**DOI:** 10.1186/1753-6561-8-S1-S32

**Published:** 2014-06-17

**Authors:** Zhao Yang, Duncan C Thomas

**Affiliations:** 1 Department of Preventive Medicine, University of Southern California, Los Angeles, CA 90089-9234, USA

## Abstract

The cost of next-generation sequencing is now approaching that of the first generation of genome-wide single-nucleotide genotyping panels, but this is still out of reach for large-scale epidemiologic studies with tens of thousands of subjects. Furthermore, the anticipated yield of millions of rare variants poses serious challenges for distinguishing causal from noncausal variants for disease. We explore the merits of using family-based designs for sequencing substudies to identify novel variants and prioritize them for their likelihood of causality. While the sharing of variants within families means that family-based designs may be less efficient for discovery than sequencing of a comparable number of unrelated individuals, the ability to exploit cosegregation of variants with disease within families helps distinguish causal from noncausal ones. We introduce a score test criterion for prioritizing discovered variants in terms of their likelihood of being functional. We compare the relative statistical efficiency of 2-stage versus1-stage family-based designs by application to the Genetic Analysis Workshop 18 simulated sequence data.

## Background

Most genome-wide association studies for discovering common variants associated with disease traits have been conducted using a case-control design with unrelated controls. Not only are unrelated individuals easier to identify and enroll than are entire families (particularly multiple-case families), but the statistical efficiency per subject genotyped is typically higher using unrelated controls than using unaffected siblings or other relatives [1]. However, with the growing interest in rare variants and the availability of next-generation sequencing technology, there has been a resurgence of interest in using family-based designs [2-5]. Althoughfamily-based designs are less efficient for discovering novel variants than designs using unrelated individuals with the same total number of subjects, they may have other advantages that may outweigh this limitation. Specifically, by exploiting information about cosegregation with disease within families, they may be more efficient at prioritizing potentially causal variants from noncausal ones for subsequent testing for association with disease in larger samples. The ability to exploit mendelian inheritance may also substantially improve the imputation of rare variants in untested samples [6]. Finally, family-based designs can exploit both between-family and within-family comparisons in various 2-stage designs for better power while being robust to bias from population stratification [7-11]. The net result could be improved power for the ultimate goal of discovering novel associations with disease. We focus here on the first of these advantages.

## Methods

### Study designs

We consider a 2-stage design using individuals from a family study for discovery of novel variants and screening, followed by association testing in an independent data set using the linear regression of the phenotype on the genotype. A subset of members is selected for sequencing, preferentially sampling those with the most extreme phenotypes. We rank all the variants identified in these individuals using a novel score test and select those most likely to be causal for genotyping and association testing in the replication data set. These designs are compared with a single-stage family-based design in which all available members are sequenced [12].

### Methods of prioritization

*Bayes factor criterion*. Following the principles described in Petersen et al [13], the probability that any particular variant is causal under a given genetic model can be estimated by accumulating likelihood ratio contributions (the ratio of the likelihoods of the data under the alternative hypothesis that a particular variant is causal to the likelihood under the null hypothesis that it is not causal) across families. The likelihood ratio requires a specific alternative hypothesis to be tested against the null hypothesis, and estimation of these parameters is likely to be highly unstable for rare variants. To avoid this, Petersen et al [13] compute a Bayes factor (BF) by averaging over a prior distribution of allele frequencies and relative risks. BFs are computed as the ratio of the conditional probabilities of the joint genotypes of the sampled individuals under the true model to that under the null.

*Score test criterion*. A simpler alternative to the BF calculations for distinguishing causal from noncausal variants was described by Ionita-Laza et al [14]. Because score tests are computed under the null hypothesis, they do not require specification of an alternative hypothesis distribution of minor allele frequencies (MAFs) and relative risks (RRs) for causal alleles. Ionita-Laza et al compute a score contribution for variants shared by each pair of relatives, based on their population frequency and degree of relationship, add these scores over all families, and compare it to an approximate null mean and variance. Here we explore an extension of this basic idea to incorporate all available phenotype information in a pedigree, including the phenotypes of subjects without sequence data. We compute the score statistic:

$$\begin{array}{ccc} T_{v} & =\Sigma_{f}t_{fv}=\Sigma_{f}\left({\text{Y}}_{f}-\mu_{f}\text{1}\right)\prime \Phi_{f}^{-1}\left({\text{G}}_{fv}-q_{v}\text{1}\right) & \\ & =\Sigma_{f}\left({\Sigma_{i}}_{\in Nf}{\Sigma_{j}}_{\in \text{S}f}\left(Y_{fi}-\mu_{f}\right)\Phi_{fij}^{-1}\left(G_{fjv}-q_{v}\right)\right) \end{array}$$

where **Y***_f _*is the vector of phenotypes for family *f*; μ*_f _*is the family-specific mean phenotype; **Φ***_f_* is the kinship matrix, that is, the matrix of kinship coefficients; **G***_fv_* is the vector of genotypes for variant *v*; and *q_v _* is its minor allele frequency. The *G_fv_*-*q_v_* deviationsare set to zero for untyped individuals, but the inclusion of the kinship terms for typed-untyped pairs allows their phenotypes to contribute. This statistic has mean zero under the null hypothesis and asymptotic variance var(*T_v_*) = ∑*_f_t_fv_*^2^. Very similar tests have recently been described by Schifano et al [15] and Chen et al [16]. For the purpose of prioritizing variants, it is sufficient to calculate the score test *T_v_*^2^/var(*T_v_*) for each variant and select the top-ranked ones at some cutoff. In other simulations, we have found this statistic to be highly correlated with the BF, to show nearly as good discrimination between causal and noncausal variants, and to be computationally much faster.

### Application to Genetic Analysis Workshop 18 data

We compared the various design and analysis alternatives on a subset of the simulated Genetic Analysis Workshop 18 (GAW18) data. Based on the "answers" provided, we chose to focus on the *MAP4 *region of chromosome 3, which contains 15 functional variants having the strongest associations with both diastolic blood pressure (DBP) (6.5% of the total phenotypic variants) and systolic blood pressure (SBP) (7.8%). These 15 variants spanned a broad range of MAFs and effect sizes, individually accounting for from 2.8% to <0.3% of the variance. We selected all the variants in the region from 200 kilobases (kb) upstream to 100 kb downstream of the transcription start site (1151 variants in total). For comparison, we selected six 300-kb regions at random from those on chromosome 3 that harbored no functional variants for either trait and included all variants in these regions (6195 variants in total). For simplicity, we used the most likely genotypes for the imputed individuals, although the expected allele dosages would have been better. We also limited our initial exploration to 1000 variants (all 15 functional and 985 of the null variants).

For each trait, we preprocessed the phenotype data using a general linear mixed model to extract the intercept and slope coefficients for age and their variances for each individual, after adjustment for gender and current hypertension treatment, with random effects for family. These estimates were then treated as the phenotypes in the genetic analysis of each variant individually, using linear regression.

For the single-stage design, we analyzed the associations using all 959 individuals using the quantitative transmission disequilibrium test with mating-type means (QTDT_M_) [17] and tabulated the proportion of functional and nonfunctional variants that were associated at 0.05 significance after Bonferroni correction. We also tried the FBAT-rare procedure [18,19], which is similar except that the test is performed at the nuclear family level rather than at the individual offspring level. Because of the small number of informative nuclear families, the variance estimator from this test was less stable. Because no residual within-family dependency was simulated, the QTDT_M _test is valid, so we present the results only from this test.

For the 2-stage design, we first selected 2, 4, or 6 members of each pedigree for whom sequencing data were available, excluding those in the subset of maximally unrelated individuals and the closely related full sib and parent-offspring pairs. We used a logistic function of the squared rank deviation from the family's median phenotype to select these members at random. We used the sequence data on only these sampled individuals (along with the complete phenotype data) to compute the score test for each variant. In the second stage, we tested the association of the top-ranked associations in the data set of unrelated individuals using linear regression of the phenotype on the genotype, with Bonferroni adjustment for only the prioritized variants. We varied the thresholds for prioritization from 0.5% to 16% of the top-ranked variants.

Because of the computational burden, we restricted these analyses to phenotype replicates 1 to 5 and analyzed each replicate using 20 random subsets of members' sequence data.

## Results

Table 1 summarizes the results for 985 null and 15 functional variants in the *MAP4 *region for DBP and SBP measurements, using the baseline observation, intercept, and slope parameters as the phenotype. The results for baseline and intercepts were generally similar and somewhat stronger for SBP than for DBP, so subsequent analyses are presented only for SBP intercepts. The mean scores showed a clear gradient in mean score statistics between negative, null, and positive variants, albeit with substantial overlap between their distributions (SDs about 1.0). Power was very low for both the 1-step and 2-step procedures, and generally the 1-step procedure yielded higher power (13.3% vs. 4.0% when restricted to the top 100 prioritized variants) despite the larger multiple testing penalty. Slope estimates showed opposite effects from intercepts and were generally very weak, as might be expected as no effects on slopes were simulated, only a shift in level. Extending this to all 6195 null variants lowered power for both 1-stage and 2-stage results, as expected, but the mean scores for the null variants were then very close to zero.

**Table 1:** Mean score tests for the complete pedigrees.

Phenotype	Parameter	Mean score test			Proportion of variants (%)					
					
					Prioritized		Replicated		1-Stage QTDT_M_	
	
	Simulated	-	Null	+	Null	+/-	Null	+/-	Null	+/-
DBP	Baseline	-1.42	-0.25	+0.42	10.2	14.7	1.8	5.3	1.7	9.3
	Intercept	-1.36	-0.32	+0.24	10.1	17.3	1.4	4.0	3.5	8.0
	Slope	+0.70	+0.14	-0.12	10.2	13.3	1.7	0.0	3.3	4.0

SBP	Baseline	-1.40	-0.35	+0.20	10.2	16.0	0.3	2.7	1.7	9.3
	Intercept	-1.34	-0.31	+0.37	10.1	17.3	0.0	4.0	2.0	13.3
	Slope	+0.85	+0.21	+0.11	10.2	12.0	0.5	0.0	2.3	0.0

Mean score tests for the complete pedigrees for protective, null, and deleterious variants, along with the proportion of the top 100 variants prioritized using only the related members and replicated at α= 0.05/100 using only the unrelated members, and the proportion of variants significant at α= 0.05/1000 in a single-stage QTDT_M _test. The "+" and "−" represent variants with positive and negative association with the phenotypes respectively. If higher blood pressure is assumed to have more risk, "+" would correspond to deleterious variants and "−" would correspond to protective variants. In total, there are 6 deleterious variants, 9 protective variants, and 961 noncausal variants being discovered and tested.

**Figure 1:**
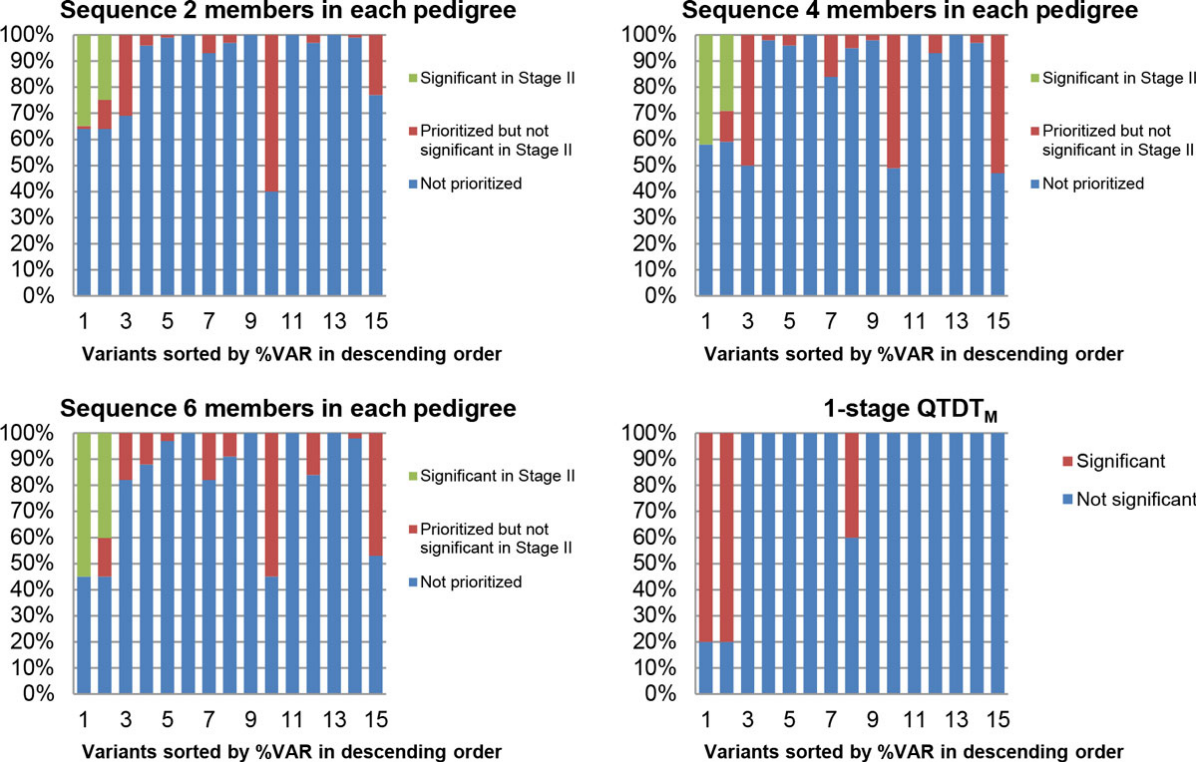
**Comparison of 1-stage and 2-stage designs**. The proportion of being prioritized and being significantfor all 15 functional variants in the *MAP4 *gene (sorted by %VAR explained in descending order):QTDT_M _for the 1-stage procedure using all members; score test for prioritizing the top 100 variants using 2, 4, or 6 randomly selected related members for sequencing and prioritizing). SBPintercepts model only.

Most of the 15 functional variants in *MAP4 *were either very rare or had weak effects. Figure 1 compares the 1-stage and 2-stage results, varying the number of individuals whose sequence data was used for prioritization. The 2 variants accounting for the largest variance (2.79% and 1.49%) were significant in the QTDT_M _using all the pedigree members in a single stage in 4 of the 5 replicates analyzed (80% power), while 1 variant with only weak effects (0.05% of variance) was significant in 2 of 5 replicates; 2 other variants with relatively large effects (1.43% and 1.11%) were not significant in any of the 5 replicates. The first and second strongest variants were prioritized 36%, 42%, and 55% of the time using 2, 4, and 6 subject's sequence data, respectively, and the majority of these were replicated. Three other variants--1 with the third largest effect and 2 with very weak effects--were prioritized with relatively large probabilities, but none were ever replicated.

## Discussion

With only 15 causal variants (most of them with very small effects) in the subset of variants we analyzed, we cannot reliably compare the power of the various designs, although for these data, the 2-stage procedure did not seem to perform better than the 1-stage procedure. This may be partly a result of the small size of the replication sample of unrelated individuals (*N *= 157). As a test, we expanded the data set by combining the unrelated individuals from 5 replicates for the second step and the power for the joint test rose substantially (results not shown). Despite the lower power of the 2-stage approach, the cost is *much *lower because only a small number of individuals need to be fully sequenced and the genotyping required for replication is much cheaper. Because the costs of sequencing and targeted genotyping are rapidly changing and could be quite different for individual- and family-based designs, we have not addressed *cost*-efficiency. See [12] and [20] for discussion of optimization of sampling fractions for studies of independent individuals using individual and pooled sequencing under cost constraints.

We arbitrarily fixed the total number of variants to be prioritized at 100, but the general principles for design of 2-stage designs [21-24] could be applied to optimize the allocation of sample sizes across the 2 stages and the threshold for prioritizing variants, subject to cost constraints. As this threshold becomes more restrictive, fewer variants will be selected, lowering power for the first stage, but because the penalty for multiple testing will be less, power for the second stage will be improved; a similar tradeoff applies to sample size allocation between the 2 stages. As a preliminary exploration, we varied this threshold between 0.5% and 16% of variants, and the overall power increased monotonically with the threshold. Of course, the number of false positives also increased, but at a much lower rate, so that the false discovery rate dropped with increasing threshold and number of members sequenced. Still, the false discovery rate is very large, so that a further replication would be needed to weed out the false positives.

It is also possible that more appropriate adjustment for time-dependent treatment data would improve power for all these analyses (although it's unlikely to affect the *relative *performance of the 1-stage and 2-stage designs). Because treatment is itself related to blood pressure, it is both a confounder and an intermediate variable on a causal pathway, so neither ignoring it nor covariate adjustment is appropriate. This problem was extensively discussed at Genetic Analysis Workshop 13 [25]; see references [26-29] for discussion of several better approaches. As a rough test of this hypothesis, we reran the analysis using the simulated effect sizes for gender and treatment, and the results (not shown) were essentially unchanged.

## Competing interests

The authors declare that they have no competing interests.

## Authors' contributions

ZY participated in statistical analysis. DCT developed the statistical method. Both authors participated in drafting and revising the manuscript. Both authors read and approved the final manuscript.
